# Biodegradation of Chloroxylenol by *Cunninghamella elegans* IM 1785/21GP and *Trametes versicolor* IM 373: Insight into Ecotoxicity and Metabolic Pathways

**DOI:** 10.3390/ijms22094360

**Published:** 2021-04-22

**Authors:** Marta Nowak, Katarzyna Zawadzka, Janusz Szemraj, Aleksandra Góralczyk-Bińkowska, Katarzyna Lisowska

**Affiliations:** 1 Department of Industrial Microbiology and Biotechnology, Faculty of Biology and Environmental Protection, University of Łódź, 12/16 Banacha Street, 90-237 Łódź, Poland; katarzyna.zawadzka@biol.uni.lodz.pl (K.Z.); aleksandra.goralczyk@biol.uni.lodz.pl (A.G.-B.); 2 Department of Medical Biochemistry, Medical University of Łódź, 6/8 Mazowiecka Street, 92-215 Łódź, Poland; janusz.szemraj@umed.lodz.pl

**Keywords:** biodegradation, chloroxylenol, cytochrome P450, detoxification, environmental xenobiotics, filamentous fungi, laccase

## Abstract

Chloroxylenol (PCMX) is applied as a preservative and disinfectant in personal care products, currently recommended for use to inactivate the SARS-CoV-2 virus. Its intensive application leads to the release of PCMX into the environment, which can have a harmful impact on aquatic and soil biotas. The aim of this study was to assess the mechanism of chloroxylenol biodegradation by the fungal strains *Cunninghamella elegans* IM 1785/21GP and *Trametes versicolor* IM 373, and investigate the ecotoxicity of emerging by-products. The residues of PCMX and formed metabolites were analysed using GC-MS. The elimination of PCMX in the cultures of tested microorganisms was above 70%. Five fungal by-products were detected for the first time. Identified intermediates were performed by dechlorination, hydroxylation, and oxidation reactions catalysed by cytochrome P450 enzymes and laccase. A real-time quantitative PCR analysis confirmed an increase in CYP450 genes expression in *C. elegans* cells. In the case of *T. versicolor*, spectrophotometric measurement of the oxidation of 2,20-azino-bis (3-ethylbenzthiazoline-6-sulfonic acid) (ABTS) showed a significant rise in laccase activity during PCMX elimination. Furthermore, with the use of bioindicators from different ecosystems (Daphtoxkit F and Phytotoxkit), it was revealed that the biodegradation process of PCMX had a detoxifying nature.

## 1. Introduction

Xenobiotics occurring in the natural environment at low concentrations are referred to as anthropogenic micropollutants (MPs), which are a wide and heterogeneous group of organic compounds that enter the natural environment as a result of human activities. Despite their low concentrations, they can disrupt ecosystem function [1]. One of the representatives of these compounds is chloroxylenol (4-chloro-3,5-dimethylphenol, para-chloro-meta-xylenol, PCMX), which is an antimicrobial agent applied in disinfectant products, such as hand washing liquids, liquid soaps, Dettol and as a preservative in human care and cosmetic products [2]. Chloroxylenol has unique antimicrobial activity and is effective against bacteria, fungi, algae, and viruses. Currently, due to the global pandemic caused by a new kind of coronavirus, severe acute respiratory syndrome coronavirus 2 (SARS-CoV-2), many cosmetics and household chemicals have been enriched with disinfectants. The National Environment Agency (NEA) in Singapore has compiled a list of active substances and concentrations effective against the virus. One of the active ingredients is chloroxylenol at a concentration of 0.12% [3,4,5].

The widespread use of such products may result in the disruption of wastewater treatment unit operations and the release of MPs into the natural environment. Wastewater treatment plants (WWTPs) with residues of xenobiotics are identified as the major route responsible for contamination with micropollutants. A maximum concentration (404 µg L^−1^) was detected in sewage treatment plants in China [6]. Despite good removal (>95%) in WWTPs, its concentration in effluent was reported to be approximately 19 to 3010 ng L^−1^ in Europe and 1700 ng L^−1^ in North America [7,8,9,10]. Incomplete removal of preservatives in WWTPs and subsequent discharge of effluents result in the presence of MPs residues in the receiving environments. Recent research regarding the occurrence of PCMX in surface waters was conducted by Dsikowitzky et al. in Jakarta city (Indonesia), where PCMX was detected in the range of 20–1200 ng L^−1^ [11,12]. PCMX was also observed in European rivers at a frequency of 20–90%, at maximum concentrations ranging from 254–358 ng L^−1^ [8,9,13].

Chloroxylenol in humans, after the long dermal exposition, is a skin and eye irritant and can modify the blood cellular composition [14]. In terms of ecotoxicity, published studies regarding the effect of PCMX on environmental species are very scarce. PCMX residue in the environment poses toxicological risks to various organisms from low to high trophic levels in aquatic environments. Recent studies on PCMX ecotoxicity have shown genotoxicity, histopathology, embryonic mortality, neurotoxicity, morphology changes and the modulation of endocrine functions in aquatic organisms. Moreover, PCMX may cause changes in the community structure of the microorganisms in the soil [15,16]. Available studies regarding the toxicological impact of PCMX on various organisms strongly suggest its potential to cause environmental health risks.

The harmful impact of chloroxylenol on the natural environment has caused the development of effective approaches for its elimination from WWTPs with the use of physicochemical and biological methods. For the elimination of chlorophenols, including PCMX, many common physicochemical approaches have been applied, including an advanced oxidation process with the use of ozonation, ultraviolet irradiation and thermally activated persulfate [17,18,19,20,21]. Despite the high efficiency of elimination and short-term treatment, there are some disadvantages, including the cost, the use of numerous chemical solvents, and the formation of harmful by-products. Biological methods, using microorganisms are inexpensive and are considered a major method of PCMX conversion in the natural environment. The biodegradation activity of natural microbial communities plays a key role in eliminating MPs, which is why increasing interest in understanding the metabolic pathways of xenobiotic degradation has been observed in recent years [22]. Products of microbial biodegradation of MPs might be more active or more toxic than parent compounds. Moreover, a mixture of different by-products may exhibit a higher toxicity than each metabolite alone. For these reasons, it is important to study how PCMX is transformed and check the toxicity of its by-products. There are many literature data describing the ability of microorganisms to eliminate chloroaromatic compounds; unfortunately, studies about PCMX are very scarce. Brahma et al. [23] isolated and characterized PCMX-resistant bacteria, which effectively eliminated the preservative with the formation of fewer toxic products. Moreover, several fungal species, especially those belonging to *Aspergillus* sp., are described as good PCMX degraders. As reported by Ghanem et al. [24], degradation rates of 99.72% of PCMX (2 mg L^−1^) by *Aspergillus niger* were observed within seven days. Information about PCMX microbial degradation pathways is still unknown. However, mammalian metabolism of PCMX was studied by Thomas et al. [25], where cytochrome P450 monooxygenases removed PCMX, mainly by side chain oxidation and aromatic hydroxylation. Moreover, *ipso* substitution was evaluated as a minor degradation pathway. Much more research concerns the biodegradation pathways of other chloroaromatic compounds. Kang et al. [26,27] reported recombinant enzyme complexes cloned from *Arthrobacter chlorophenolicus* and *Escherichia coli* which catalysed the elimination of 4-chlorophenol. The side chain monooxygenation during the degradation of chlorotoluenes by toluene dioxygenase from *Pseudomonas putida* and by chlorobenzene dioxygenase from *Burkholderia* sp. was observed by the team Lehning et al. [28]. Likewise, the ability of *Comamonas testosterone* to degrade chlorotoluenes was investigated. The increase in the activity of catechol 1,2-dioxygenase, responsible for the *ortho* cleavage pathway, was observed [29]. The metabolism of chlorobenzenes via hydroxylation, where chlorine atoms were substituted by hydroxyl groups, and via the *ortho* ring cleavage pathway, were noted in the *Bacillus subtilis* strain [30]. Furthermore, the enzymes of the *ortho* cleavage pathway were involved in the metabolism of toluene by *Burkholderia fungorum* [31]. In contrast, during the biodegradation of 2-chlorotoluene by *Rhodococcus* sp., 4-chlorophenol by mixed microbial community via *meta* cleavage pathway were involved [32,33]. With respect to the fungi, the ability to biodegrade chloroaromatic compounds was widely investigated. The involvement of cytochrome P450 monooxygenases catalysing the degradation of chlorophenols through hydroxylation and dehalogenation was observed [34]. The removal of 2,4-dichlorophenol by *Thielavia* sp. was conducted by the formation of hydroxylated and oxidized metabolites [35]. Furthermore, ligninolytic enzymes were found to play a key function in the degradation of chlorophenols. Kordon et al. [36] reported the total dechlorination of monochlorinated hydrobiphenyl by the laccase of *Pycnoporus cinnabarinus* and *Myceliophthora thermophila*.

In this study, we focused on the ability of the fungi *Cunninghamella elegans* IM 1785/21GP and *Trametes versicolor* IM 373 to remove chloroxylenol and proposed its degradation by-products. Furthermore, we suggested enzymes involved in this process and determined the toxicity of PCMX untreated and treated with *C. elegans* and *T. versicolor* strains.

## 2. Results and Discussion

### 2.1. Growth and Chloroxylenol Elimination

The conducted analyses revealed a high tolerance of both strains towards high concentrations of chloroxylenol after 120 h of incubation (Figure S1). Only at the highest concentration of PCMX (50 mg L^−1^) was a statistically significant inhibition of *T. versicolor* (77%) and *C. elegans* (86%) growth observed. Tolerance to chloroxylenol seems to be microorganism- and PCMX concentration-specific, although the majority of reports point to the impact of this preservative on the inhibition of growth. In the work by Gomaa et al., the percent inhibition of growth of *Candida albicans* rose from 3.9% to 100% with increasing the PCMX concentration from 10–70 ppm [37]. An inverse relationship between the PCMX concentration and fungal growth was reported for *Aspergillus niger* [24]. In the study of Mohammed and Al-Jibouri, most of the tested fungal growth was significantly inhibited by chloroxylenol at a concentration of 1.25%, but in the case of *Aspergillus flavus*, *Mucor* and *Fusarium*, a higher tolerance was observed [38].

Among the available scientific literature, researchers mention the physicochemical losses of PCMX rather than biodegradation. For treating PCMX-bearing waste streams, several advanced oxidation processes (AOPs) have been identified, including UV/O3, sonochemical, and electrochemical methods. Scientists have also focused on the elimination of PCMX from WWTPs by sorption and coagulation. Many of these approaches have outstanding short-term treatment efficiencies, but all of them have significant limitations and disadvantages, such as costly infrastructure and higher by-product toxicity [20,21,39]. Available literature data concerning the biological treatment of PCMX in WWTPs suggest that PCMX in the conventional activated sludge system is not decreased to satisfactory levels that avert the ecological risk to receiving bodies of water [40]. The described methods apply mainly to processes taking place under controlled conditions, e.g., in sewage treatment plants. However, we do not know much about the processes occurring in natural conditions with the participation of microorganisms. *Cunninghamella elegans* and *Trametes versicolor* are well known for their ability to eliminate different hazardous pollutants, including drugs, pesticides, dyes and personal care products [41,42,43,44]. Despite the fact that chloroxylenol is a micropollutant occurring in the natural environment, there is still little information available about its elimination and fate. An initial quantitative analysis showed that the elimination of the preservative was closely correlated with the growth of mycelium (Figure S1). Both tested species were able to efficiently remove PCMX from culture medium supplemented with the preservative concentration in the range 5–25 mg L^−1^, where the inhibition of growth was not observed. The level of elimination in these cultures ranged from 63% to 79% (*p* < 0.05). The highest residue of PCMX (100%), with reference to the abiotic control, was in the samples with the initial concentration of 50 mg L^−1^, where the highest growth limitation of the tested fungi was noted. The variant containing 25 mg L^−1^ chloroxylenol was selected for the next stages of experiments, because PCMX at the chosen concentration was eliminated by the tested fungi with the highest efficiency.

**Figure 1:**
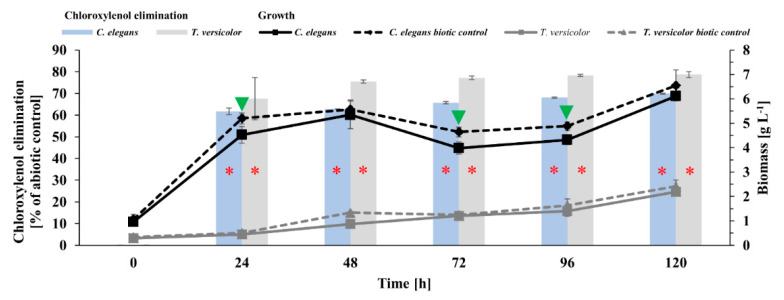
Kinetics of chloroxylenol elimination and the growth of *C. elegans* and *T. versicolor* cultures incubated with the addition of PCMX at a concentration of 25 mg L^−1^ on Czapek-Dox medium supplemented with 20 g L^−1^ glucose. Each result represents an average ± SD (*n* = 6). Statistical analyses were performed using the Mann–Whitney U test (* *p* < 0.05—a statistically significant increase in PCMX elimination relative to the abiotic control; ▼ *p* < 0.05—a statistically significant decrease in biomass in the sample with PCMX relative to the biotic control).

The intensity of *C. elegans* and *T. versicolor* growth in the presence of the chosen PCMX concentration is demonstrated in Figure 1. Biotic controls were incubated as reference samples for the same time periods. After 120 h of cultivation, the tested concentration (25 mg L^−1^) of PCMX did not cause significant differences in the amounts of *T. versicolor* and *C. elegans* biomass between samples with PCMX and controls.

The dynamics of preservative elimination by *C. elegans* and *T. versicolor* cultures (Figure 1) showed a rapid decrease in the amount of the xenobiotic. The removal of PCMX (25 mg L^−1^) started 24 h after its supplementation, and only 33% and 39% of PCMX were detected in the *T. versicolor* and *C. elegans* extracts, respectively. Among the tested strains, *T. versicolor* was characterized by higher effectiveness than *C. elegans*. After 120 h, the residues of the preservative in those cultures were 21% and 30%, respectively. Some research has also focused on identifying microorganisms effective towards PCMX biodegradation, for instance, a study conducted for PCMX elimination by the fungi *Aspergillus* spp. An efficient degradation level was obtained by *Aspergillus niger*, which conducted an elimination process for 7 days with a yield of 99.72% at 2 mg L^−1^ PCMX. An increase in the PCMX concentration from 2 mg L^−1^ to 20 mg L^−1^ led to a decrease in the degradation yield by approximately 8% [24]. The team of Ghanem et al. also assessed the ability of *Aspergillus terreus* and *Aspergillus versicolor* to eliminate PCMX [24]. The studied strains degraded only 55.62% and 45.62% of PCMX (2 mg L^−1^). In another study regarding the optimization of the PCMX elimination process with the use of different methods of immobilization of *Klebsiella pneumoniae* 2D, the results revealed that immobilization on polyurethane foam pieces was the most effective in degrading PCMX, showing the highest percentage of elimination (88.3%) after 24 h of incubation. The elimination results obtained for free cells reached 52% [45]. A microbial population in the activated sludge was tested for the possibility of eliminating PCMX, which occurred at different concentrations. The obtained results showed that the level of biodegradation significantly depended on the PCMX input to the sewage. Eighty-seven percent removal was achieved for 0.5 mg L^−1^ and 44% for 5 mg L^−1^ [40].

### 2.2. Identification of PCMX Biodegradation Metabolites

**Figure 2:**
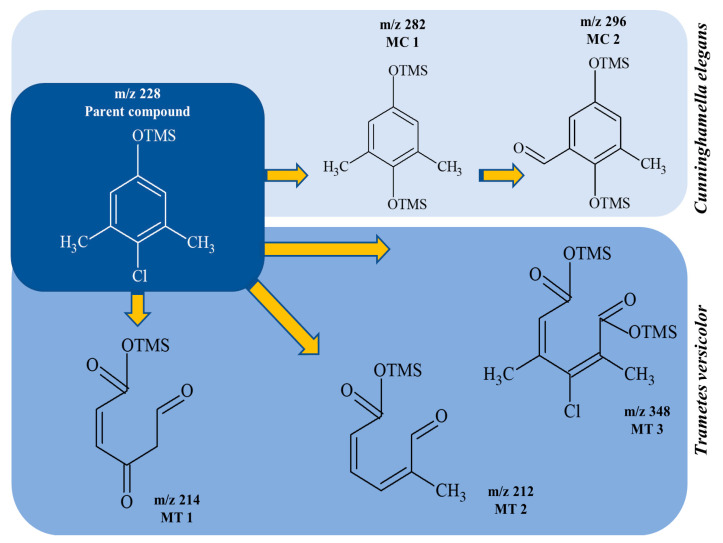
The chemical structure of chloroxylenol by-products formed by the tested strain.

Despite the few reports on the elimination of chloroxylenol by microorganisms, no data are currently available on what and how microorganisms degrade PCMX. Therefore, the present study focuses not only on the ability of *C. elegans* and *T. versicolor* to eliminate PCMX, but also on PCMX detoxification by formed metabolites and enzymatic mechanisms involved in this process. Qualitative analyses of PCMX metabolites formed by *T. versicolor* and *C. elegans* were carried out in 24 h incubation periods until the end of the experiment. After extract derivatization with BSTFA, the samples were analysed with GC-MS. None of the intermediates were detected in the biotic and abiotic controls. The first step of qualitative analysis was the determination of the fragmentation pattern of BSTFA-derivatized chloroxylenol. It was identified based on a molecular ion [M]^+^ at *m/z* 228 for trimethylsilylation (TMS) derivatives and a retention time (Rt) of 7.323 min. The fragment ion at *m/z* 213 was observed after the loss of methyl [M-15]^+^ from the trimethylsilyl group. The ion at *m/z* 177 was obtained due to the loss of methyl groups and further loss of the chloride atom from the molecular ion of chloroxylenol. The ion at *m/z* 73, which corresponded to the TMS derivative fragment ion [(CH_3_)_3_Si]^+^, suggested that the hydroxyl group in the molecule was efficiently derivatized. In the next step, a search for chloroxylenol by-products formed by *C. elegans* and *T. versicolor* was carried out. The structure of the detected compounds was assigned from the fragmentation pattern and *m/z* values obtained. In our research, PCMX metabolites formed during the biotransformation process conducted by microorganisms were identified (Table 1). PCMX biotransformation by *C. elegans* probably occurred via dehalogenation, aromatic ring hydroxylation and methyl group oxidation, with the formation of two metabolites, which were identified as 2,6-dimethylbenzene-1,4-diol, di-TMS (MC1), and 2,5-dihydroxy-3-methylbenzaldehyde, di-TMS (MC2). The results of the analysis of its peak area are presented in Supplementary Materials (Figure S3). The compound MC1 showed a molecular ion [M]^+^ at *m/z* 282 for TMS derivatives and an Rt of 8.67 min with fragment ions at *m/z* 267 (M^+^-CH_3_), 223 (M^+^-2CH_3_), 193 (M^+^-OTMS) and 73 (TMS). The metabolite was detected for the first time after 24 h of incubation, and the analysis of its peak area showed an increase in its amount within the fungal culture. The compound MC2 had an Rt of 8.733 min, a molecular ion [M]^+^ at *m/z* 296 for TMS derivatives, and characteristic fragment ions at *m/z* 281, 252, 179 and 73, and was detected at 24 h of incubation only. In the case of the *T. versicolor* strain, three acids formed by ring-opening of the parent compound during dehalogenation; hydroxylation and oxidation were detected. Peak MT1 with an Rt of 6.435 min was supposed to be 4,6-dioxohex-2-enoic acid, TMS, which showed positive ion chemical ionization at [M]^+^ at *m/z* 214 for TMS derivatives and fragmentation ions at *m/z* 143, 129, 75, and 73. The second identified product, MT2 (Rt 6.696 min), was observed to be 5-methyl-6-oxohexa-2,4-dienoic acid, TMS with a molecular ion [M]^+^ at *m/z* 212 and ion fragments at *m/z* 197, 183, 169, and 73. The molecular weight of the compound with Rt 15.501 min (MT3) was [M]^+^ at *m/z* 348. Based on the molecular weight and its fragmentation ions (259, 217, 147, 73), the compound was identified as 3-chloro-2,4-dimethylhexa-2,4-dienedioic acid, di-TMS. The metabolite MT1 was detected for the first time after 48 h of incubation and MT2 and MT3 after 24 h of incubation. The analysis of the peak area of *T. versicolor* metabolites showed an increase in their amount within the fungal culture, with maximum concentrations on the last day of incubation. The proposed chemical structure of the detected derivatives is shown in Figure 2.

Due to the lack of information about microbial biotransformation of PCMX, we compared our results to reports on mammalian metabolism and physicochemical degradation. Among the methods of chloroxylenol removal and complete mineralization, the most popular is degradation with the use of AOPs. Li et al. proposed the degradation pathway of PCMX in UV or UV/persulfate systems [20]. The use of these methods leads to aromatic ring-opening reactions, dechlorination and oxidation, resulting in the formation of short-chain carboxylic acids and total mineralization. Similar results were obtained by Sun et al., who conducted PCMX degradation by thermally activated persulfate in aqueous solution [21]. Chloroxylenol biotransformation through the substitution of a chlorine atom by a hydroxyl group (as in MC1) was described for mammalian liver microsomes [25]. It was also detected that side chain oxidation (methyl group hydroxylation) led to the production of mono- and dehydroxylated metabolites. The authors suggested that PCMX hydroxylation was a common biotransformation process conducted by cytochrome P450 in higher organisms. Some reports have documented the ability of fungi to degrade several halogenated pollutants, i.e., alkylphenols and chlorophenols. The degradation process proceeded mainly via hydroxylation and halogenation reactions. Mtibaá et al. evaluated the ability of the ascomycetes *Thielavia* sp. HJ22 to remove phenolic xenobiotics, i.e., 2,4-dichlorophenol was found to be metabolized by the formation of hydroxylated and oxidized derivatives [35].

### 2.3. Mechanisms of Chloroxylenol Biotransformation

**Figure 3:**
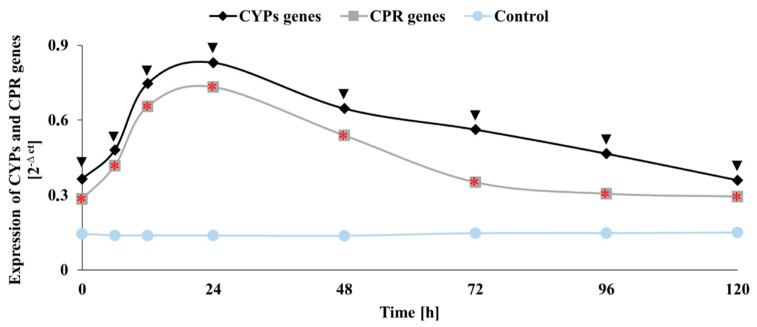
The expression of cytochrome P450 and cytochrome P450 reductase genes in the cells of *C. elegans* incubated with the addition of PCMX at a concentration of 25 mg L^−1^ compared to the control without the xenobiotic. Each result represents an average ± SD (*n* = 3). Statistical analysis was performed using the Mann–Whitney U test (▼ *p* < 0.05—a statistically significant increase in CYP450s genes relative to the biotic control; * *p* < 0.05—a statistically significant increase of CRP genes relative to the biotic control).

*Cunninghamella* sp. has the ability to biotransform pharmaceuticals and other xenobiotics by oxidation and conjugation reactions, forming compounds similar to those found in humans and other mammals. These fungi have been widely investigated as xenobiotic metabolism models in mammals [43]. In our work, we analysed the activity of CYP450 and cytochrome reductase (CRP) genes to evaluate the participation of these monooxygenase systems in the biodegradation of PCMX by *C. elegans*. We also studied the influence of CYP450 inhibitors on the elimination process yield. Among the tested strains, CYP450 was involved in the metabolism of chloroxylenol only in the case of *C. elegans*. The profile of by-products emerging in the *C. elegans* culture suggested the involvement of these enzymes. Three CYP450 inhibitors, sodium azide, 1-aminobenzotriazole and proadifen, caused a significant inhibition of PCMX degradation reactions (*p* < 0.05) (Figure S2). After a 120 h incubation of the fungus with inhibitors, the remaining amount of PCMX reached approximately 96, 82, and 82%, respectively. In the case of the cultures incubated with metyrapone, no statistically significant suppression was observed; nonetheless, a high residue (79%) of PCMX compared to the abiotic control was also detected. A less visible inhibition of elimination was noted in the cultures of *T. versicolor* incubated with CYP450 inhibitors and PCMX. In this case, the efficiency of PCMX elimination reached 65, 60, 76 and 66% in the samples, with the addition of sodium azide, 1-aminobenzotriazole, metyrapone and proadifen, respectively, compared to the abiotic control (Figure S2). These results suggest that the CYP450 enzyme system might not have been involved in the elimination process in the fungus *T. versicolor*. To confirm the participation of the CYP450 enzyme system in the PCMX elimination process in *C. elegans* cultures, the real-time quantitative PCR of CYP450s and CRP genes was also performed (Figure 3). An increase in CYP450s and CRP genes expression was observed after 6 h of cultivation, and its maximum was reached after 24 h of incubation. Then, a decrease in their expression levels was noted, while in the biotic sample, no changes in the expression during the whole time period of the experiment were detected. The greatest expression of CYP450s and CRP genes in 24 h of incubation was correlated with the high elimination of PCMX during the first 24 h in the *C. elegans* culture. Furthermore, a less visible increase in the efficiency of elimination after 24 h to the end of the experiment corresponded with a decrease in CYP450s and CRP gene expression. The MC1 metabolite (Table 1) was probably formed analogically to the formation of metabolites with rat, mouse, and human microsomes. CYP450 enzymes can remove para-substituents from phenols with an *ipso* substitution pathway, which involves the formation of a p-benzoquinone derivative with the loss of a halide ion, and then reduced to the final hydroquinone component [25]. Similar studies were conducted by Zawadzka et al. for *C. elegans* fungi, which were incubated with carvedilol. An increase in the levels of CYP450s and CRP genes and inhibition of the xenobiotic elimination by supplementation with CYP450 inhibitors were also shown. Furthermore, the profile of carvedilol biotransformation fungal products (hydroxylated and conjugative metabolites) indicated that CYP450 enzymes were involved in the process [42]. Monooxygenases can also play a role in the degradation of chlorophenols through successive hydroxylation and dehalogenation reactions, according to Arora and Bae [34]. The formation of hydroxylated by-products during the biodegradation process of 2,4-dichlorophenol in *Thielavia* sp. HJ22 is also catalysed by cytochrome P450 monooxygenases [35].

**Figure 4:**
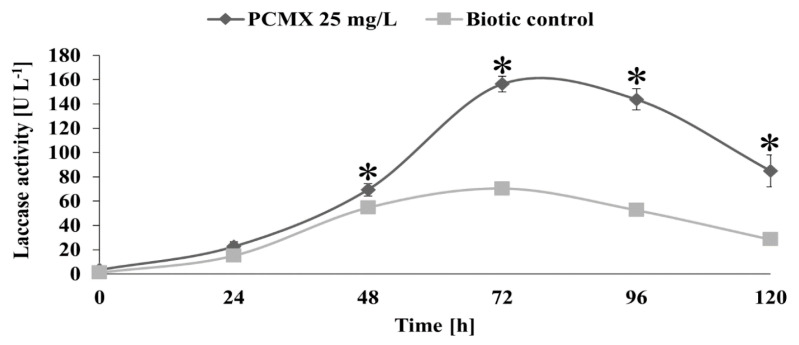
Laccase activity in *T. versicolor* cultures incubated with the addition of PCMX at a concentration of 25 mg L^−1^ for 120 h. Each result represents an average ± SD (*n* = 3). Statistical analysis was performed using the Mann–Whitney U test (* *p* < 0.05—a statistically significant increase in laccase activity relative to the control).

White-rot fungi (WRF), including *T. versicolor*, are mostly used in the degradation of a wide variety of pollutants, as well as those found in personal care products. WRF contain nonspecific oxidative extracellular enzymes like laccase, which belongs to the class of multicopper oxidases [46]. This group of enzymes in fungi catalyses the degradation process by oxidatively cleaving the aromatic ring present in the lignin structure of wood, giving fungi the ability to degrade toxic xenobiotics. Laccases can catalyse the oxidation of various substrates, including phenols [47]. The next stage of the study involved evaluating the influence of PCMX on ligninolytic enzyme activity and the contribution of these enzymes to the elimination of the preservative. The production of laccase by *T. versicolor* occurred in 24 h and reached its maximum at 72 h (156.4 U L^−1^) for the cultures supplied with PCMX. At the same time period, the activity of laccase in the control samples was 70.4 U L^−1^ (Figure 4). In the cultures older than 72 h, the enzyme activity decreased, and in 120 h of incubation, laccase activity was detected until 84.9 U L^−1^. Laccase activity stimulation by different xenobiotics is well-documented. Mougin et al. reported increased laccase activity in a *T. versicolor* culture supplemented with different compounds of industrial origin (e.g., aniline, nonylphenol, diquox or 9-fluorenone) [48]. Increased activity of this enzyme during the biodegradation of bisphenol A and diclofenac by *T. versicolor* was noted by Yang et al. [49]. By-products formed in *T. versicolor* cultures suggest the involvement of the ligninolytic enzyme in the biodegradation process of PCMX. Unfortunately, very little is known about the pathways of PCMX and other chlorophenols transformation by filamentous fungi, which play an important role in ecosystems. Our results present a total dechlorination of PCMX, which may be caused by oxidative release of chloride. Some previous studies reported that laccase plays a key function in the enzymatic oxidation of contaminants including phenolic compounds. A similar reaction was described by Kordon et al., who revealed total dechlorination of monochlorinated hydroxybiphenyl by the laccase of *Pycnoporus cinnabarinus* and *Myceliophthora thermophila*, which led to the formation of less toxic derivatives [36]. Navada and Kulal reported chloramphenicol dehalogenation and oxidation catalysed by laccase, resulting in the formation of chloramphenicol aldehyde, which was non-toxic to microorganisms [50]. The formation of MT1, MT2, and MT3 products (Table 1) could also be obtained through two pathways: direct oxidation of a product catalysed by laccase, and reductive dehalogenation of a product catalysed primarily by CYP450 monooxygenases, followed by an oxidative reaction catalysed by laccase. Further research will be needed to fully elucidate the mechanisms involved in the process of PCMX biodegradation by filamentous fungi.

### 2.4. Toxicity Assessment

Chloroxylenol is a well-known antimicrobial chemical with a long history of use, however information about its ecotoxicology is very scarce. Moreover, the fate of PCMX in the natural environment is unknown. Therefore, the ecotoxicity estimation of PCMX and its by-products formed by microorganisms in ecosystems is necessary. Metabolites formed by biodegradation are a major concern of ecotoxicology because of a potential harmful effect on organisms that is lower or higher than that of the parent compound. However, microbial biodegradation of toxic chemicals involving dechlorination, hydroxylation, oxidation, and conjugation reactions leads to the detoxification of the substrate [16,51]. To evaluate the toxicity changes during the biotransformation of PCMX by *C. elegans* and *T. versicolor*, Daphtoxkit F and Phytotoxkit bioassays were applied. The ecotoxic effects of filtrates from fungal cultures were assessed using bioindicator species from different ecosystems, i.e., *Daphnia magna* as a freshwater species and *Lepidium sativum* and *Sorghum saccharatum* as soil, water and waste contaminant indicators. The results of the toxicological studies are presented in Table 2. Moreover, the 48 h LC_50_ (half maximal lethal concentration) of PCMX for crustaceans and 72 h EC_50_ (half maximal effective concentration) of PCMX for plants were evaluated. The LC_50_ value for *D. magna* was estimated at 8.78 mg L^−1^, and the EC_50_ values for *L. sativum* and *S. saccharatum* were established at 30.79 mg L^−1^ and 15.4 mg L^−1^, respectively. Slight toxicity of the biotic controls of both fungi was observed for all tested organisms. The toxicity of the abiotic control was constant during the time course of the experiment. Czapek–Dox medium did not have a toxic effect on the tested organisms. A comparison of the percentage effect for the tested samples from 0 h of incubation and xenobiotic-treated fungal samples from 120 h indicated a reduction in toxicity. Biotransformation of PCMX by *C. elegans* caused a decrease in toxicity from 84 ± 8.7% to 45.2 ± 5.2% and from 91.1%. ± 6.4% to 37.7 ± 9.5% and from 100% to 20 ± 16.3% in *L. sativum*, *S. saccharatum* and *D. magna*, respectively. Likewise, in the case of *T. versicolor* culture, the biodegradation process led to a reduction in the inhibition of root growth by three times and more than two times in *L. sativum* and *S. saccharatum* biotests, respectively. The mortality of *D. magna* after 48 h of incubation with postculture liquids from *T. versicolor* achieved 100% (0-day of incubation) and 40 ± 23.1% (5-day of incubation), which meant a 2.5-fold reduction in toxicity. Similar results had been obtained in our previous report, where we did not observe an increase in the toxicity of methylisothiazolinone derivatives after incubation with *Phanerochaete chrysosporium* [52]. Additionally, Zawadzka et al. showed a decrease in the toxicity of metabolites of carvedilol formed by *Cunninghamella echinulate* [51]. Sun et al. evaluated PCMX degradation by AOPs, which led to the formation of more toxic compounds during the initial stages of oxidation. However, the authors pointed out that the next stages of degradation by thermally activated persulfate resulted in the formation of less toxic intermediates than those formed by the parent compound [21].

## 3. Materials and Methods

### 3.1. Chemicals

Chloroxylenol, metyrapone, proadifen hydrochloride, 1-aminobenzotriazole, sodium azide, 2,20-azino-bis (3-ethylbenzthiazoline-6-sulfonic acid) (ABTS), and N, O-bis(trimethylsilyl)trifluoroacetamide (BSTFA) were obtained from Sigma-Aldrich (Poznań, Poland). High purity grade ethyl acetate and 96% ethanol were purchased from POCH S.A. (Gliwice, Poland). All PCR reagents came from Applied Biosystems (Foster City, CA, USA). All the other chemicals were from Sigma-Aldrich (Darmstadt, Germany), POCH S.A. (Gliwice, Poland) and Chempur (Piekary Śląskie, Poland).

### 3.2. Microorganisms and Culture Conditions

The strains *Cunninghamella elegans* IM 1785/21GP and *Trametes versicolor* IM 373 originated from the Microorganisms Collection of the Department of Industrial Microbiology and Biotechnology, University of Lodz, Poland. Spores from 14-day-old ZT slants [52] were used to inoculate 20 mL Sabouraud medium (BD Difco, Franklin Lakes, NJ, USA) supplemented with 2% glucose in 100 mL Erlenmeyer flasks. Cultivation was carried out on a rotary shaker (140 rpm) at 28 °C. After 1 d of incubation, the preculture of *C. elegans* was transferred to fresh medium at a ratio of 1:2 and incubated for the next 24 h. The preculture of *T. versicolor* was carried out for 3 days. The homogenous precultures of *C. elegans* and *T. versicolor*, prepared as presented above, were transferred into modified Czapek–Dox medium (NaNO_3_ 1 g; KH_2_PO_4_ 1 g; KCl 0.5 g; MgSO_4_ × 7H_2_O 0.5 g; FeSO_4_ × 7H_2_O 0.01 g; glucose 20 g up to 1 L; pH 6.6) at a ratio of 1:9. The concentrations of PCMX in the culture medium were 5, 10, 25, and 50 mg L^−1^. To evaluate the elimination kinetics of PCMX, the expression of cytochrome P450 and cytochrome P450 reductase genes, laccase activity and toxicity, only one concentration (25 mg L^−1^) of PCMX was used. Biotic controls (without the xenobiotic) and abiotic controls (without fungal inoculum) were also prepared. Moreover, proadifen, metyrapone, 1-aminobenzotriazole and sodium azide, nonselective CYP450 inhibitors, were added to some cultures at concentrations of 1, 2.5, 4 and 1 mM for *C. elegans* and 0.5, 1, 1, and 0.1 mM for *T. versicolor*. CYP450 inhibitors and PCMX stocks were prepared in 96% ethanol except sodium azide, which was diluted in distilled water. The cultures were incubated on a rotary shaker (120 rpm for *C. elegans* and 140 rpm for *T. versicolor*) at 28 °C. Samples were collected over 5 days at different time intervals. For biomass assessment, the mycelia were separated by Whatman filter paper number 1 (Sigma-Aldrich, Darmstadt, Germany), washed twice with distilled water and dried at 100 °C, until a constant weight was achieved.

### 3.3. Quantitative Analysis of PCMX

The whole content of each *C. elegans* and *T. versicolor* culture after incubation was homogenized mechanically with the use of a Mixer Mill MM400 (Retsch, Haan, Germany) with glass beads (1 mm diameter). Next, 20 mL of each homogenate was extracted for 10 min with the same volume of ethyl acetate. The collected organic phases were dried over anhydrous Na_2_SO_4_. The residues after evaporation to dryness under reduced pressure at 40 °C were dissolved in 2 mL ultrapure ethyl acetate, diluted, and transferred to chromatography vials for quantitative analyses. Extraction efficiency was 87 ± 5.2%. The PCMX concentration was determined using a Hewlett-Packard HP 6890 gas chromatograph and HP 5973 mass spectrometer. An HP 5 MS capillary column (Agilent, 30 m × 250 µm, film thickness 0.25 µm) was used for the chromatographic separation of PCMX. The injection volume was 1.6 µL. The inlet was set to split mode with a split ratio of 10:1, and the split flow was 12 mL min^−1^. The temperature was kept at 280 °C. The carrier gas was helium at a steady flow rate of 1.2 mL min^−1^. The temperature of the column was maintained at 80 °C for 2 min, and then it was increased at a rate of 20 °C min^−1^ to 300 °C and maintained for 3 min. The total time of analysis was 16 min. Selective ion monitoring (SIM) mode was targeted at quantifying ions at *m/z* 121 and qualifier ions at *m/z* 156. A quantitative analysis was performed using a calibration curve within the working range from 2.5 to 50 µg L^−1^ PCMX.

### 3.4. Qualitative Analysis of PCMX Elimination

For the identification of chloroxylenol metabolites, the obtained extracts were dissolved in 1 mL of ethyl acetate and evaporated to dryness. Next, 50 µL of BSTFA was appended and warmed up to 60 °C for 60 min, according to the method described by Krupiński et al. and Nowak et al. [53,54]. Then, the samples were supplemented with 450 µL of ethyl acetate and diluted. GC-MS analysis was conducted in scan mode, with the mass range set from 25 amu to 450 amu. The injection volume was 1.6 µL. All PCMX by-products were identified on the basis of the mass spectra analysis.

### 3.5. Analysis of Cytochrome P450 and Cytochrome P450 Reductase Genes

*C. elegans* cultures with and without chloroxylenol at a concentration of 25 mg L^−1^ were collected for 5 days for the analysis of CYP450 and CRP gene expression according to the method described in a previous publication [42]. The InviTrap Spin Universal Kit (Stratec Molecular, Berlin, Germany) was used for total RNA isolation. A Picodrop spectrophotometer was used to assess the total RNA concentration. An Agilent RNA 6000 Nano Kit (Agilent Technologies, Santa Clara, CA, USA) and 2100 Bioanalyzer (Agilent Technologies, Santa Clara, CA, USA) were used to verify the quality of isolated total RNA.

cDNA from extracted total RNA of *C. elegans* was obtained with the TaqMan^®^ RNA Reverse Transcription Kit (Applied Biosystem, Foster City, CA, USA) according to the manufacturer’s instructions. Primers for RT-PCR were constructed on the basis of the Lisowska et al. paper [45]. TAMRA dye and FAM reporter dye were used to label the 3′ and 5′ ends of the sequence, respectively. The ABI Prism 7000 Sequence Detection System (Applied Biosystems, Foster City, CA, USA) was used to quantify the cytochrome P450, cytochrome P450 reductase and GAPDH genes. All samples were incubated at 50 °C for 2 min and at 95 °C for 10 min and then cycled at 95 °C for 30 s, 60 °C for 30 s and 72 °C for 1 min for 40 cycles. Fluorescence emission data were collected, and mRNA levels were quantified using a critical threshold (Ct) value. Relative gene expression levels were obtained using ΔCt standard 2^−Δct^ calculations [55].

### 3.6. Determination of Laccase Activity

Laccase activity in liquid cultures was determined during 5 days of cultivation using supernatant obtained after culture centrifugation for 10 min at 10,000 rpm. Laccase activity was assayed according to the method described earlier by Góralczyk-Bińkowska et al., with slight modifications [56]. The measurement of ABTS oxidation was monitored spectrophotometrically at 420 nm. The reaction mixture (2 mL) contained 1800 µL citrate phosphate buffer (pH 4.5), 100 µL of the tested sample and 100 µL of 10 mM ABTS. The sample without culture supernatant was used as a reference blank. The concentration of the enzyme needed to oxidize 1 M substrate per minute was specified as one unit of laccase activity (U).

### 3.7. Toxicity Assays

*Daphnia magna* Daphtoxkit F magna (Microbiotests, Inc., Mariakerke-Gent, Belgium) was used according to the manufacturer’s procedure and ISO 6341 standard. *D. magna* ephippia were incubated in standard fresh water for 72 h at 20 °C at 6000 l×. The motile larvae were subjected to an acute toxicity test. The cultures of *C. elegans* and *T. versicolor* with or without PCMX (control) were separated by filtration. The supernatant and abiotic samples containing chloroxylenol at the same concentration as the biotic samples were sterilized by filtration through sterile Sartorius membrane filters (0.25 µm pore size). The obtained supernatants and Czapek–Dox medium were diluted twice in standard fresh water. PCMX stock solution, at appropriate concentrations, was added to fresh water to evaluate the LC_50_. The standard fresh water was used as a growth control. The toxicity of PCMX and its metabolites was calculated as a percentage of dead larvae after 48 h of incubation.

The assessment of *C. elegans* and *T. versicolor* postculture liquids and PCMX phytotoxicity was performed using *Lepidium sativum* and *Sorgum saccharatum* seeds. The evaluation of root growth was conducted with a Phytotoxkit (Microbiotests, Inc., Mariakerke–Gent, Belgium) in accordance with the instructions and ISO 18763 standard. Briefly, commercially available seeds were rinsed with sterile deionized water and placed in test plates containing a layer of filter paper moisturized with 20 mL of the tested sample prepared as described above. A stock solution of PCMX was diluted with sterile deionized water, which was used as a control. The test plates were covered with the bottom part of the plate and placed in an incubator in a vertical position for 72 h at 25 °C (±1 °C) in darkness. At the end of the incubation period, the percentage of root length inhibition was calculated, and the EC_50_ was evaluated.

### 3.8. Data Analysis

Statistica 13.3 software was used to do the statistical analyses (StatSoft, Kraków, Poland). Sample variability was given as a standard deviation (±SD). The Mann–Whitney U test was used to investigate the statistical significance. Values of *p* < 0.05 were considered significant.

## 4. Conclusions

The ability of *C. elegans* and *T. versicolor* to remove 70% and 79% of PCMX within 120 h of incubation, was shown. Moreover, five metabolites of PCMX formed by oxidation, hydroxylation, and dehalogenation reactions, were identified. The involvement of two different groups of enzymes, including intracellular CYP450 and extracellular laccase, was revealed. This report also demonstrated the detoxification of PCMX by the tested microorganisms via the reduction of the xenobiotic concentration and the formation of less toxic metabolites. The results of the present study indicate how PCMX could be metabolized in the natural environment and show the possibility of using the tested fungi as potential tools for environmental bioremediation.

## Figures and Tables

**Table 1:** Gas chromatography-mass spectrometry results of qualitative analysis of chloroxylenol degradation by *C. elegans* and *T. versicolor*.

ID	RT (min)	Proposed Compound Name	Chemical Formula/Structure	Molecular Mass (Da)	Mass Spectrum *m/z* (10 Largest Ions Relative Abundance)	Proposed Fragmentation Pattern EI-MS *m/z* [Ion]
Parent compound	7.323	4-chloro-3,5-dimethylphenol, TMS	C_11_H_17_ClOSi 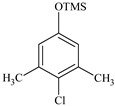	228.79	73 (21.4), 91 (12), 93 (16), 177 (17.6), 213 (99.9), 214 (17.5), 215 (36.7), 228 (61.9), 229 (11.6), 230 (22.7)	228 [C_11_H_17_ClOSi^+^] 215 [C_10_H_16_ClOSi^+^] 213 [C_10_H_14_ClOSi^+^] 177 [C_10_H_13_OSi^+^] m/z 73 [C_3_H_9_Si^+^]
*Cunninghamella elegans*						
MC1	8.670	2,6-dimethylbenzene-1,4-diol, di-TMS	C_14_H_26_O_2_Si_2_ 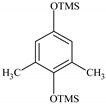	282.1	45 (11.1), 73 (67,9), 126 (13), 193 (60.9), 194 (11), 223 (72.1), 224 (16.1), 267 (99.9), 268 (23.7), 282 (24.5)	282 [C_14_H_26_O_2_Si_2_^+^] 267 [C_13_H_23_O_2_Si_2_^+^] 223 [C_10_H_15_O_2_Si_2_^+^] 193 [C_11_H_17_OSi^+^] 73 [C_3_H_9_Si^+^]
MC2	8.733	2,5-dihydroxy-3-methylbenzaldehyde, di-TMS	C_14_H_24_O_3_Si_2_ 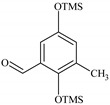	296.1	45 (10.3), 73 (99.9), 74 (8.6), 75 (20.8), 81 (12.4), 164 (12.3), 179 (26.8), 252 (12.4), 281 (13.8), 296 (12.8)	296 [C_14_H_24_O_3_Si_2_^+^] 281 [C_13_H_21_O_3_Si_2_^+^] 252 [C_12_H_20_O_2_Si_2_^+^] 179 [C_10_H_15_OSi^+^] 73 [C_3_H_9_Si^+^]
*Trametes versicolor*						
MT1	6.435	4,6-dioxohex-2-enoic acid, TMS	C_9_H_14_O_4_Si 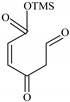	214.76	28 (20.3), 45 (19), 56 (18.2), 73 (99.9), 75 (54.9), 129 (30.3), 130 (23.1), 143 (46.7), 157 (44), 214 (33.9)	214 [C_9_H_14_O_4_Si^+^] 143 [C_6_H_7_O_4_^+^] 129 [C_5_H_9_O_2_Si^+^] 75 [C_2_H_7_OSi^+^] 73 [C_3_H_9_Si^+^]
MT2	6.696	5-methyl-6-oxohexa-2,4-dienoic acid, TMS	C_10_H_16_O_3_Si 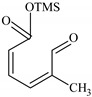	212.36	73 (99.9), 75 (76.2), 127 (17.7), 141 (17.7), 169 (93.3), 170 (32.7), 183 (18.6), 184 (25), 197 (19.6), 212 (25.1)	212 [C_10_H_16_O_3_Si ^+^] 197 [C_9_H_13_O_3_Si^+^] 183 [C_8_H_11_O_3_Si^+^] 169 [C_7_H_9_O_3_Si^+^] 73 [C_3_H_9_Si^+^]
MT3	15.501	3-chloro-2,4-dimethylhexa-2,4-dienedioic acid, di-TMS	C_14_H_25_ClO_4_Si_2_ 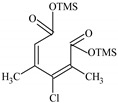	348.70	73 (99.9), 103 (21.5), 129 (18), 147 (31.8), 157 (9.9), 217 (49), 218 (10.1), 228 (24), 259 (40.4), 348 (14.5)	348 [C_14_H_25_ClO_4_Si_2_^+^] 259 [C_11_H_16_ClO_3_Si^+^] 217 [C_9_H_21_O_2_Si_2_^+^] 147 [C_5_H_15_OSi_2_^+^] 73 [C_3_H_9_Si^+^]

**Table 2:** Toxicity analysis of *C. elegans* and *T. versicolor* cultures with chloroxylenol in the Daphtoxkit F and phytotoxicity assays. Each result represents an average ± SD (*n* = 4). Statistical analysis was performed using the Mann–Whitney U test with * *p* < 0.05.

Percentage Effect [%]						
	*L. sativum* 72 h		*S. saccharatum* 72 h		*D. magna* 48 h	
Sample	0 h	120 h	0 h	120 h	0 h	120 h
*C. elegans* + PCMX	84.0 ± 8.7 *	45.2 ± 5.2 *	91.5 ± 6.4 *	37.7 ± 9.5	100.0 ± 0.0 *	20.0 ± 16.3
*C. elegans*	20.5 ± 12.1	36.1 ± 7.5 *	20.5 ± 15.5	25.0 ± 13.6	5.0 ± 10.0	20.0 ± 23.1
*T. versicolor* + PCMX	100.0 ± 0.0 *	32.9 ± 8.4	100.0 ± 0.0 *	41.5 ± 12.9	100.0 ± 0.0 *	40.0 ± 23.1 *
*T. versicolor*	35.2 ± 16.1 *	38.8 ± 24.3 *	41.5 ± 18.9	45.3 ± 20.8	15.0 ± 19.2	20.0 ± 23.1
Abiotic control	81.3 ± 11.4 *	77.6 ± 19.6 *	94.3 ± 7.2 *	84.9 ± 18.5 *	90.0 ± 11.6 *	100.0 ± 0.0 *

## Supplementary Materials

The following are available online at https://www.mdpi.com/article/10.3390/ijms22094360/s1, Figure S1: Chloroxylenol elimination and the growth of *C. elegans* and *T. versicolor* cultures after 120 h of incubation with PCMX at concentrations of 5, 10, 25 and 50 mg L^−1^. Figure S2: Effect of cytochrome P450 inhibitors on chloroxylenol elimination by *C. elegans* and *T. versicolor*., Figure S3: The peak area of detected metabolites of PCMX produced by *C. elegans* (A) and *T. versicolor* (B) during 120 h of incubation.
