# Supplementary material for: Biodegradation of Chloroxylenol by Cunninghamella elegans IM 1785/21GP and Trametes versicolor IM 373: Insight into Ecotoxicity and Metabolic Pathways
Source: Int J Mol Sci. 2021 Apr 22;22(9):4360. doi: 10.3390/ijms22094360 (PMC8122528; doi:10.3390/ijms22094360)
Supplement: Supplementary file 1 [file ijms-22-04360-s001.zip › ijms-1170882-supplementary.pdf]

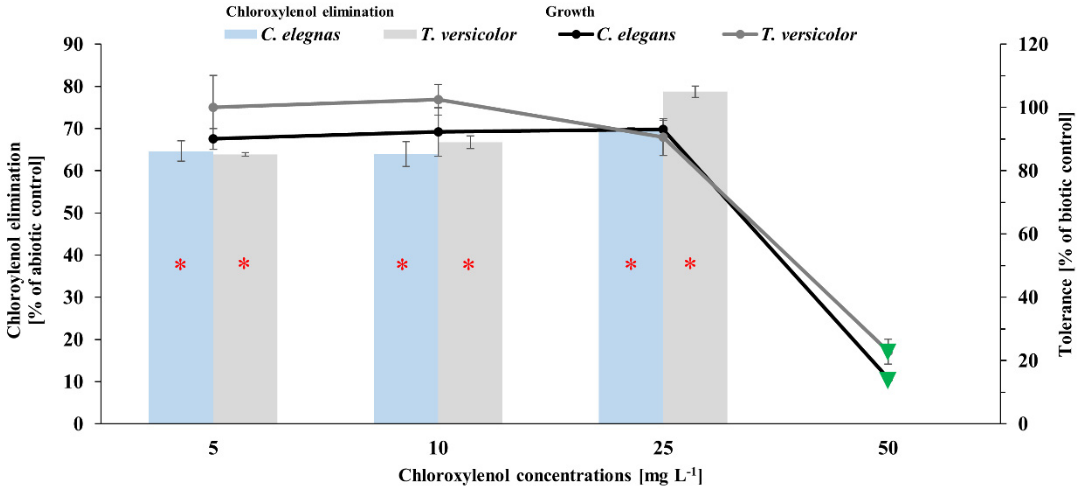

Figure S1. Chloroxylenol elimination and the growth of *C. elegans* and *T. versicolor* cultures after 120 h of incubation with PCMX at concentrations of 5, 10, 25 and 50 mg L^{-1}. Each result represents an average ±SD (n = 6). Statistical analyses were performed using the Mann-Whitney U test (*$p < 0.05$ – a statistically significant increase in PCMX elimination relative to the abiotic control; ▼ $p < 0.05$ – a statistically significant decrease in biomass in the sample with PCMX relative to the biotic control).

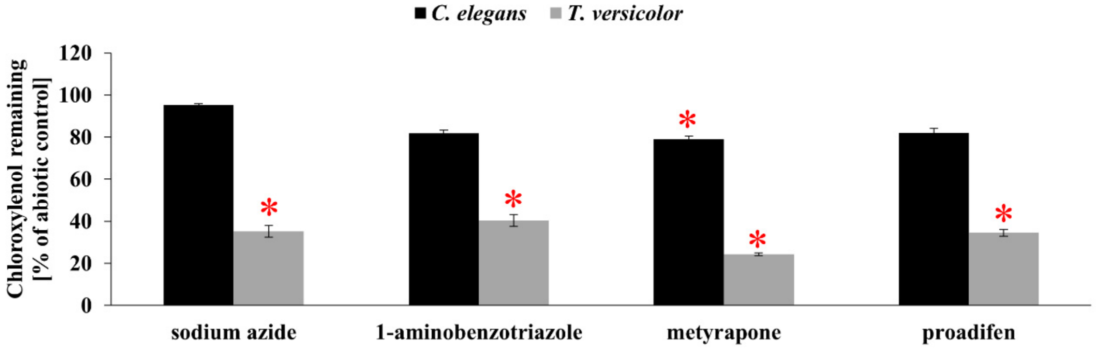

Figure S2. Effect of cytochrome P450 inhibitors on chloroxylenol elimination by *C. elegans* and *T. versicolor*. Each result represents an average ± SD (n = 6). Statistical analysis was performed using the Mann-Whitney U test with *$p <$ 0.05.

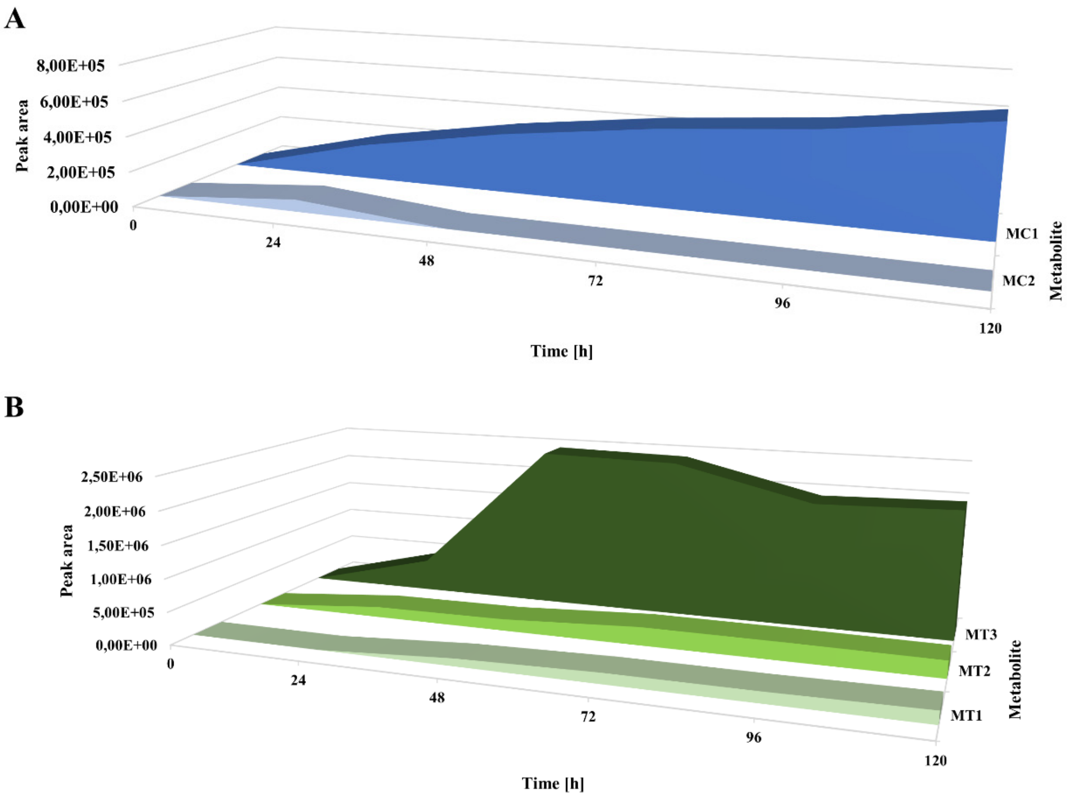

Figure S3. The peak area of detected metabolites of PCMX produced by *C. elegans* (**A**) and *T. versicolor* (**B**) during 120 h of incubation.
